# Distinct tumour antigen-specific T-cell immune response profiles at different hepatocellular carcinoma stages

**DOI:** 10.1186/s12885-021-08720-9

**Published:** 2021-09-08

**Authors:** Chaoran Zang, Yan Zhao, Ling Qin, Guihai Liu, Jianping Sun, Kang Li, Yanan Zhao, Shoupeng Sheng, Honghai Zhang, Ning He, Peng Zhao, Qi Wang, Xi Li, Yanchun Peng, Tao Dong, Yonghong Zhang

**Affiliations:** 1 Biomedical Information Center, Beijing YouAn Hospital, Capital Medical University, Beijing, China; 2 Interventional Therapy Center of Liver Disease, Beijing YouAn Hospital, Capital Medical University, Beijing, China; 3 Clinical Laboratory Center, Beijing YouAn Hospital, Capital Medical University, Beijing, China; 4 MRC Human Immunology Unit, MRC Weatherall Institute of Molecular Medicine, Oxford University, Oxford, UK; 5 CAMS Oxford Institute, Nuffield Department of Medicine, Oxford University, Oxford, UK; 6 Department of Radiology and Research Institute of Radiology, The Affiliated Cancer Hospital of Zhengzhou University, Henan Cancer Hospital, Zhengzhou, Henan China

**Keywords:** Cancer-testis antigen, Distinction, Hepatocellular carcinoma, T cell immune response, Tumour-associated antigen

## Abstract

**Background:**

Cancer-testis antigens (CTAs) and tumour-associated antigens (TAAs) are frequently expressed in hepatocellular carcinoma (HCC); however, the role of tumour-antigen-specific T cell immunity in HCC progression is poorly defined. We characterized CTA- and TAA-specific T cell responses in different HCC stages and investigated their alterations during HCC progression.

**Methods:**

Fifty-eight HCC patients, 15 liver cirrhosis patients, 15 chronic hepatitis B patients and 10 heathy controls were enrolled in total. IFN-γ ELSPOT using CTAs, including MAGE-A1, MAGE-A3, NY-ESO-1, and SSX2, and two TAAs, SALL4 and AFP, was performed to characterize the T-cell immune response in the enrolled individuals. The functional phenotype of T cells and the responsive T cell populations were analyzed using short-term T-cell culture.

**Results:**

T cell responses against CTAs and TAAs were specific to HCC. In early-stage HCC patients, the SALL4-specific response was the strongest, followed by MAGE-A3, NY-ESO-1, MAGE-A1 and SSX2. One-year recurrence-free survival after transcatheter arterial chemoembolization plus radiofrequency ablation treatment suggested the protective role of CTA-specific responses. The four CTA- and SALL4-specific T cell responses decreased with the progression of HCC, while the AFP-specific T cell response increased. A higher proportion of CD4+ T cells specific to CTA/SALL4 was observed than AFP-specific T cell responses.

**Conclusions:**

The IFN-γ ELISPOT assay characterized distinct profiles of tumour-antigen-specific T cell responses in HCC patients. CTA- and SALL4-specific T cell responses may be important for controlling HCC in the early stage, whereas AFP-specific T cell responses might be a signature of malignant tumour status in the advanced stage. The application of immunotherapy at an early stage of HCC development should be considered.

**Supplementary Information:**

The online version contains supplementary material available at 10.1186/s12885-021-08720-9.

## Introduction

Hepatocellular carcinoma (HCC) is the fourth most common cause of cancer-related death and ranks sixth in incidence worldwide [1]. The incidence of HCC is particularly serious in China, and over 50 % of global newly diagnosed liver cancer cases and liver cancer-related deaths occur in China [2]. Therefore, there is an urgent need for effective HCC therapies, including those targeting antigens expressed by HCC as a result of tumour occurrence.

Host immunosurveillance, which plays an important role in tumorigenesis by eliminating tumour cells and suppressing tumour growth, was proposed by Paul Ehrlich a century ago [3, 4]. Several studies have shown that the immune system plays an important role in the occurrence and development of HCC [5, 6]. The function of the immune system changes during the development of HCC. Cytotoxic T lymphocytes, which target HCC tumour cells, are especially important regulators of tumour progression and protect HCC patients [7, 8]. Recently, immune checkpoint inhibitor-based immunotherapy for HCC [9, 10] has not only provided additional evidence supporting the role of the immune system in controlling HCC progression but also revealed that our understanding of the T cell immune response to HCC is insufficient, especially in terms of diverse T cell immunity in different stages of HCC.

The tumour antigens recognized by T cells have not been well characterized and may be immunogenic neoantigens that have not yet been identified in HCC. However, several cancer testis antigens (CTAs) whose expression is limited to cancer cells and reproductive tissues and is not found in adult somatic tissue can spontaneously induce a T cell response in HCC patients. CTAs comprise a range of self-derived proteins, such as melanoma-associated antigen A1 (MAGE-A1), MAGE-A3, New York esophageal squamous cell carcinoma antigen 1 (NY-ESO-1), and synovial sarcoma X break point gene 2 (SSX2), that can become immunogenic in HCC either by mutation or aberrant expression. These are currently popular and widely investigated CTAs in the field of HCC; however, the data on their involvement in HCC are insufficient [11–13]. In addition to CTAs, tumour-associated antigens (TAA) are also enriched (but not specific) in cancer cells [14]. Sal-like protein 4 (SALL4) is a type of TAA; although SALL4 is not expressed in the majority of normal human tissues, it is expressed in human embryonic stem cells, testes and ovaries, is highly expressed in HCC and is associated with aggressive HCC [15]. Alpha-fetoprotein (AFP) is derived from embryonic endoderm tissue cells. The content of AFP is high during foetal development and gradually decreases to the level observed in adults after birth. The majority of HCC patients have high levels of this antigen. Other malignant tumours of the stomach and pancreas are also often accompanied by a small number of increased AFPs [16]. Targeting CTA and/or TAA using vaccination strategies has been suggested because of their frequent expression in a large proportion of HCC cells (i.e.: MAGE-A1 and A3: > 50%; NY-ESO-1 > 30%, and SSX2 > 70%) [11, 17, 18].

Very few researchers have performed detailed and combined analyses of important tumour antigen-specific T cell responses and their associations with different HCC statuses. To address this issue, 98 individuals, including healthy controls (HCs) and those with different stages of HCC, liver cirrhosis (LC), or chronic hepatitis B virus infection (CHB), were enrolled. Overlapping peptides were synthesized to perform comprehensive T cell response analysis, covering CTAs (MAGE-A1, MAGE-A3, NY-ESO-1, SSX2) and TAAs (SALL4 and AFP). The goal of this study was to further clarify the diverse characteristics of tumour antigen-specific T cells among different HCCs.

## Patients and methods

### Patients and samples

In total, 98 individuals from Beijing YouAn Hospital were recruited, including 58 HCC, 15 LC, 15 CHB, and 10 HC individuals. The inclusion criteria were as follows: 1) diagnosis of HCC; 2) age between 18 and 75 years; and 3) patients who were unsuitable for or unwilling to receive surgery and were assessed as able to tolerate transcatheter arterial chemoembolization (TACE) or/and radiofrequency ablation (RFA) as a palliative or curative therapy. The exclusion criteria were as follows: 1) other malignancies; 2) severe coagulation disorders; 3) secondary liver cancer; 4) other immune-related diseases; and 5) any immunotherapy. The diagnostic criteria of HCC were applied according to the European Association for the Study of the Liver–European Organization for Research and Treatment of Cancer Clinical Practice Guidelines: Management of Hepatocellular Carcinoma [19], and HCC was classified based on the Barcelona Clinic Liver Cancer (BCLC) staging system [20]. At our interventional therapy centre, TACE combined with ablation therapy is the best option among the available interventional treatment strategies and is more effective than TACE or ablation treatment alone [21, 22]. We have performed many studies, and this strategy has become the standard therapy applied by our team [23–26]. Forty-one of the 58 HCC patients who were evaluated as suitable for TACE combined with RFA therapy received curative treatments and were followed up with every 3 months for 1 year. All 41 HCC patients underwent dynamic contrast enhancement CT scans to evaluate recurrence [27]. In addition, the diagnosis of LC and CHB was made according to previously reported guidelines [28, 29]. The study, conforming to the tenets of the 1990 Declaration of Helsinki, was approved by the Institutional Review Board of Beijing YouAn Hospital. Written informed consent was obtained from all candidates. Ten millilitres of blood from each patient was collected, and PBMCs were isolated by density gradient centrifugation.

### Synthetic peptides for T-cell analysis

A total of 334 overlapping peptides (18-mers overlapping by 10 amino acids) spanning the complete amino acid sequence of SALL4, MAGE-A1, MAGE-A3, NY-ESO-1, SSX2 and AFP were utilized. Their purities were determined to be > 90% by analytical high-performance liquid chromatography. Peptides were dissolved in dimethylsulfoxide (Sigma, Haverhill, Suffolk, UK) and diluted with RPMI 1640 before being combined into nine pools with 23–45 peptides per pool (Table S1).

### Human IFN-γ ELISPOT assay

As described previously [30], a total of 250,000 PBMCs with 8 μg/mL peptide per well containing RPMI 1640 medium with 10% FCS were used in a standard human IFN-γ ELISPOT assay. In brief, assays were carried out in 96-well MultiScreen filter plates (Millipore) coated with 15 mg/mL anti–IFN-γ mAb (1-DIK; Mabtech). Phytohaemagglutinin (10 μg/mL) was used as a positive control. Plates were incubated for 16–18 h. The plate was washed 5 times, and biotin-conjugated anti-human IFN-γ Ab (Mabtech, Nacka, Sweden) was added and reacted for 2 h. After washing the plate 5 times, streptavidin-ALP (Mabtech, Nacka, Sweden) was added and reacted for 1 h. Finally, newly prepared NBT/BCIP solution (Bio-Rad, Hercules, CA) was added for colour development after washing. The reaction was stopped by washing with distilled water, and the plate was dried at room temperature. Spot enumeration was performed with a CTL ELISPOT reader system (Cellular Technology Ltd., S6 Universal, America). To quantify antigen-specific responses, mean spots of the negative control were subtracted from the reaction wells, and the results were expressed as spot-forming units (SFUs) per 10^6^ PBMCs. Responses were regarded as positive if the results were at least three times the mean of the negative control wells and above 25 SFUs/10^6^ PBMCs. If background wells were 25 SFUs/10^6^ PBMCs or positive control wells were negative, the results were excluded from further analysis.

### Generation of tumour antigen specific T-cell lines

According to the IFN-γ ELISPOT results and the remaining samples, PBMCs from 5 HCC subjects were stimulated with the corresponding responsive antigen. Overlapping peptides were added to 200,000 cells for stimulation for 1 h and then the cells were grown in 96-well plates. Short-term T cell lines were grown for 10 days in AIM-V + 10% human AB serum (Invitrogen, Carlsbad, CA) supplemented with 100 μg/mL (final concentration) interleukin (IL)-2 (R&D Systems, Minneapolis, MN). In total, 19 antigen-specific T cell lines were generated.

### Intracellular cytokine staining

For intracellular cytokine staining, PBMCs were stimulated with the corresponding pooled peptide at a final concentration of 8 μg/mL for 1 h at 37 °C. Then, the cells were incubated for an additional 4 h with 1 μg/mL GolgiPlug (Brefeldin A, BD) and 0.7 μg/mL GolgiStop (Monensin, BD) and surface stained with CD107a-PE-CF594 (BD Bioscience). Unstimulated cells were used as negative controls. A combination of 50 ng/mL phorbol-12-myristate-13-acetate and 1 μg/mL ionomycin (both Sigma-Aldrich, Seelze, Germany) was used as a positive control. Dead cells were stained with LIVE/DEADR Fixable Aqua dye (Invitrogen). Surface markers, including CD3-AF700 (BioLegend), CD4-FITC (BD Bioscience), and CD8-APC-H7 (BD Bioscience), were stained. Cells were then washed and permeabilized using Cytofix/Cytoperm (BD Bioscience). Subsequently, the cells were washed with Perm/Wash buffer (BD Bioscience), stained intracellularly with Tumour Necrosis Factor (TNF)-α-APC (BioLegend), IFN-γ-BV786 (BD Bioscience), and IL-2-PE (BioLegend), fixed with 1 × CellFix solution (BD Biosciences) and acquired immediately on a BD LSR Fortessa. The data were analysed using FlowJo (Tree Star Inc., Ashland OR).

### Statistical analysis

Continuous variables are expressed as the mean ± standard deviation (SD). Statistical analysis of the data was performed using the χ^2^ test for constituent ratio analysis. Two-tailed Student’s t tests were used to compare parametric continuous data, and the Mann-Whitney U test was used when data were not normally distributed. Statistical significance was set at *P* < 0.05. Analyses were performed with SPSS software v25 (IBM, New York, USA), and graphs were constructed with GraphPad Prism 8.0 (GraphPad software Inc).

## Results

### CTA and TAA–specific T cell responses were detected in HCC patients

A total of 98 individuals were enrolled in the study: 58 had HCC, 15 had LC, 15 had CHB, and the remaining 10 were HCs. All HCC patients were classified according to the BCLC staging system. The tumour burden was significantly different among patients with different stages of HCC. Patients with early-stage (BCLC-0/A) HCC were more likely than patients with advanced-stage (BCLC-B/C) HCC to have solitary tumour lesions (84% vs. 39.39%, *P* = 0.001), small tumour volumes (76% vs. 30.3%, P = 0.001), and no vascular invasion/metastasis (100% vs. 54.55%, *P* < 0.0001). AFP and protein induced by vitamin K absence or antagonist-II (PIVKA-II), two HCC biomarkers, were analysed, and the PIVKA-II value of early-stage patients was lower than that in advanced-stage patients [84.50 (23.00, 374.25) mAU/mL vs. 512 (60.50, 4051.50) mAU/mL, *P* = 0.005] (Table 1).

**Table 1:** Characteristics of enrolled individuals

	controls			HCC	
	HC (*n* = 10)	CHB (*n* = 15)	LC (*n* = 15)	0 + A (*n* = 25)	B + C (*n* = 33)
Gender (Male/Female)	7/3	9/6	11/4	19/6	30/3
Age	51.39 ± 1.49	49.56 ± 12	48.33 ± 9.11	52.53 ± 5.80	51.82 ± 11.82
HBV/HCV/other	/	/	/	21/3/1	29/3/1
HBV-DNA (positive/negative/ND)	/	8/7/0	5/10/0	4/6/15^b^	9/10/14^b^
Tumour number (solitary/multiple)	/	/	/	21/4	13/20
Tumour volume (S/H) ^c^	/	/	/	19/6	10/23
VI/M (no/yes)	/	/	/	25/0	18/15
Differentiation (well/moderate/poor/ND)	/	/	/	2/2/3/18	1/6/4/22
Curative therapy (no/yes)	/	/	/	3/22	17/16
Recurrence at 1-year (no/yes)	/	/	/	11/11	5/11
WBC (10^9^/L)	5.22 ± 1.26	6.18 ± 2.29	4.83 ± 1.88	4.34 ± 1.63^b^	5.26 ± 2.39
HGB (g/L)	141.29 ± 11.48	147.17 ± 21.97	154.33 ± 18.49	141.42 ± 19.39	141.16 ± 19.67
PLT (10^9^/L)	221.43 ± 72.04	199.17 ± 70.41	128.42 ± 73.48^a, b^	103.88 ± 48.64^a, b^	154.66 ± 90.71
ALT (U/L)	N.D.	35.13 ± 26.50	42.29 ± 43.37	32.17 ± 23.26	48.88 ± 38.08
AST (U/L)	N.D.	41.07 ± 47.89	43 ± 31.51	36.30 ± 17.38	42.92 ± 23.08^b^
TBiL (μmmol/L)	N.D.	16.49 ± 6.08	33.16 ± 23^b^	21.02 ± 12.41	17.90 ± 9.15
ALB (g/L)	N.D.	44.78 ± 3.31	42.34 ± 5.94	38.32 ± 5.58^b^	39.82 ± 4.91^b^
PT (s)	N.D.	N.D.	15.27 ± 4.65	13.10 ± 1.61	12.37 ± 1.26
PTA (%)	N.D.	N.D.	70.33 ± 17.88	80.17 ± 13.54	86.97 ± 11.53
AFP (ng/mL)	/	/	/	169.21 ± 298.08	8729.39 ± 31,115.08
PIVKA-II (mAU/mL)	/	/	/	355.73 ± 603.26	3574.86 ± 7725.33

Abbreviations: HC, healthy control. CHB, chronic hepatitis B. LC, liver cirrhosis derived from CHB. HBV, hepatitis B virus; VI/M, vascular invasion/metastasis; ND, not determined. WBC, White Blood Cell. HGB, hemoglobin. PLT, platelet. ALT, alanine aminotransferase. AST, aspartate aminotransferase. TBil, total bilirubin. ALB, albumin. PT, prothrombin time. PTA, prothrombin activity. AFP, alpha-fetoprotein. PIVKA-II, protein induced by vitamin K absence or antagonist-II

Data were expressed as mean ± SD. ^a^: p < 0.05 compared with the HC group. ^b^: p < 0.05 compared with the CHB group. ^c^: we divided the patients into two groups according to the tumour volume, S indicates tumour volume < 15 cm^3^ and H means the tumour volume > 15 cm^3^

**Fig. 1:**
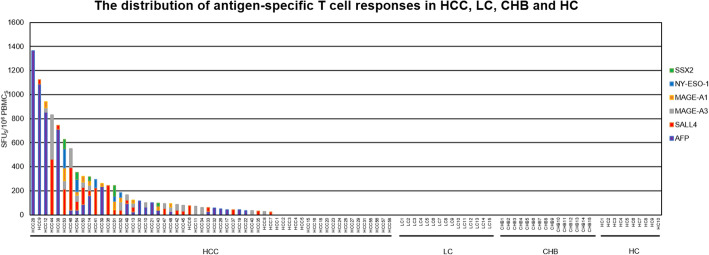
The distribution of CTA and TAA-specific T cell responses in HCC, LC, CHB and HC. CTA and TAA-specific T cell responses specific to AFP (purple), SALL4 (red), MAGE-A3 (grey), MAGE-A1 (orange), NY-ESO-1 (blue) and SSX2 (green) in 58 HCC patients, 15 LC patients, 15 CHB patients and 10 HC controls. The magnitude of T cell response was evaluated with SFU/10^6^ PBMCs in vertical coordinates (y axis), and 98 candidates, including HCC patients, LC patients, CHB patients and Healthy Control (HC), were labelled in horizontal ordinate (x axis)

CTA- and TAA-specific T cell responses were detected by IFN-γ ELISPOT assays in all 98 individuals to evaluate the comprehensive T cell response and its specificity. In total, 67.24% (39/58) of HCC patients responded to at least one CTA or TAA. In contrast, no positive response was found in any individual in any control group (LC, CHB, and HC) (Fig. S1). The difference between HCC and all control individuals was significant (*P* < 0.0001) (Fig. 1). Among the 58 HCC patients studied, 37.93% (22/58) responded to SALL4, 32.76% (19/58) to MAGE-A3, 23.40% (11/47) to MAGE-A1, 10.64% (5/47) to NY-ESO-1, 10.64% (5/47) to SSX2, and 37.93% (22/58) to AFP.

### Distinct profiles of CTA- and TAA-specific T cell responses in patients with different HCC clinical characteristics

**Fig. 2:**
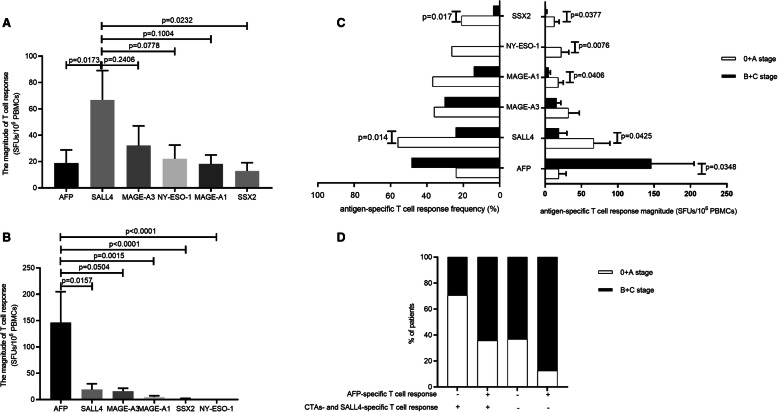
Distinct profiles of CTA/SALL4 and AFP-specific T cell response in different stages of HCC. The magnitude of T cell response against indicated antigens (including AFP, SALL4, MAGE-A1, MAGE-A3, NY-ESO-1 and SSX-2) in HCC patients with 0/A (25 subjects) (**A**) or B/C (33 subjects) stage (**B**). The T cell response magnitude (right) and the recognition frequency (left) to each kind of tumour antigen in HCC patients with BCLC-0/A (white, 25 subjects) and BCLC-B/C stage (black, 33 subjects) were presented and compared in (**C**). (**D**), Proportion of stage of HCC patients in two distinct tumour antigen specific T cell response combinations. CTAs & SALL4-specific T cell response was defined as “+” if one antigen was recognized by PBMCs at least. Non-parametric test was used to compare the response magnitude of patients in different stages, and chi-square test was used to compare the recognition frequencies

The analysis of the distribution of T cell responses revealed distinct patterns of CTA- and TAA-specific T cell responses among patients with different stages of HCC. Among patients with early-stage HCC, the strongest response was against SALL4 (66.88 ± 22.23 SFUs/10^6^ cells), followed by MAGE-A3 (32.16 ± 14.95 SFUs/10^6^ cells), NY-ESO-1 (22.21 ± 10.32 SFUs/10^6^ cells), MAGE-A1(18.21 ± 6.79 SFUs/10^6^ cells) and SSX2 (12.84 ± 6.38 SFUs/10^6^ cells), while the AFP-specific T cell response was relatively low (only 18.88 ± 10.01 SFUs/10^6^ cells). The difference between the SALL4-specific and AFP-specific T cell responses in these patients was significant (*P* = 0.0173) (Fig. 2A). Among patients with advanced-stage disease, the AFP-specific T cell response was the strongest (146.18 ± 58.75 SFUs/10^6^ cells), and much higher than SALL4 (19.15 ± 10.87 SFUs/10^6^ cells, *P* = 0.0157), MAGE-A3 (16 ± 5.63 SFUs/10^6^ cells, *P* = 0.0504), MAGE-A1 (4.93 ± 2.42 SFUs/10^6^ cells, *P* = 0.0015), SSX2 (1.21 ± 1.22 SFUs/10^6^ cells, *P* < 0.0001) and NY-ESO-1 (0, *P* < 0.0001) responses (Fig. 2B).

Interestingly, the magnitude and frequency of CTA- and SALL4-specific T cell responses was decreased in patients with advanced-stage HCC compared to those with early-stage HCC, although not all differences reached statistical significance. On the other hand, the AFP-specific T cell response showed a trend of increasing with the progression of HCC (Fig. 2C). Further analysis of the T cell response profile was therefore performed comparing early and advanced stages of HCC. The results showed that the combination of a positive CTA- and SALL4-specific T cell response and a negative AFP-specific T cell response was present in the majority of early-stage HCC patients. In contrast, a combination of a negative CTA- and SALL4-specific T cell response with a positive AFP-specific T cell response was observed in most patients with advanced-stage HCC (Fig. 2D). This result highlighted the potential protective role of CTA- and SALL4-specific T cell responses in HCC patients with early-stage disease [13].

**Fig. 3:**
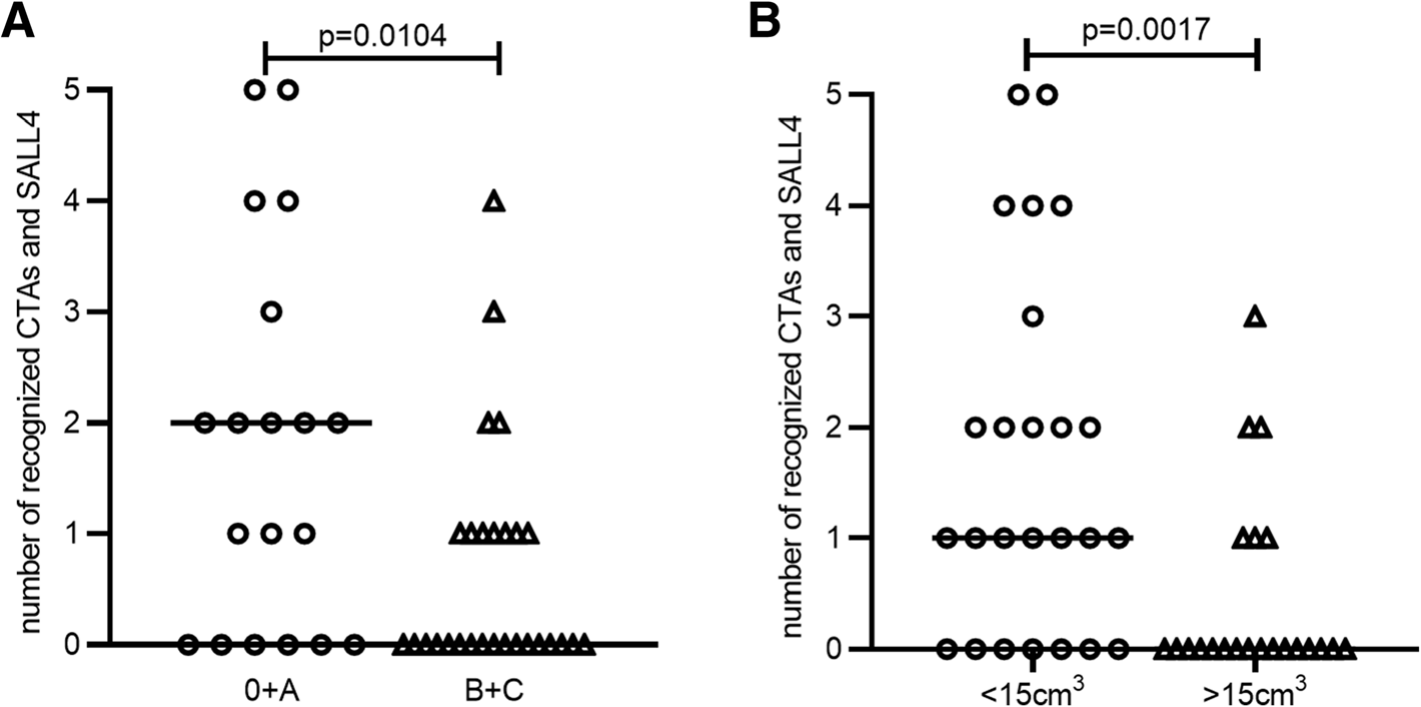
Breadth of CTA/SALL4-specific T cell response. The number of recognized individual CTA & SALL4 is shown for HCC patients according to the stage (25 subjects in 0/A stage and 33 subjects in B/C stage) (**A**) and the tumour volume (25 subjects with <15cm^3^ and 33 subjects with >15cm^3^) (**B**). Each dot represents one patient. Values were compared by Mann-Whitney U-test

Further analyses of the correlations between the breadth of the T cell response, i.e., the number of CTA, SALL4 and AFP being recognized, and the tumour stage and other tumour characteristics were performed according to the distinct profile of the tumour antigen-specific T cell response. The association between tumour stage and the breadth of CTA- and the SALL4-specific T cell response was analyzed, and more CTA- and SALL4-specific T cells were detectable in patients with early-stage HCC than in those with advanced-stage HCC (*P* = 0.0104, Fig. 3A). In addition, a comparison of the frequency of CTA-specific T cells between different tumour burdens showed that the breadth of recognition in patients with a low tumour burden (< 15 cm^3^) was broader than that in patients with a high tumour burden (> 15 cm^3^, *P* = 0.0017, Fig. 3B).

**Fig. 4:**
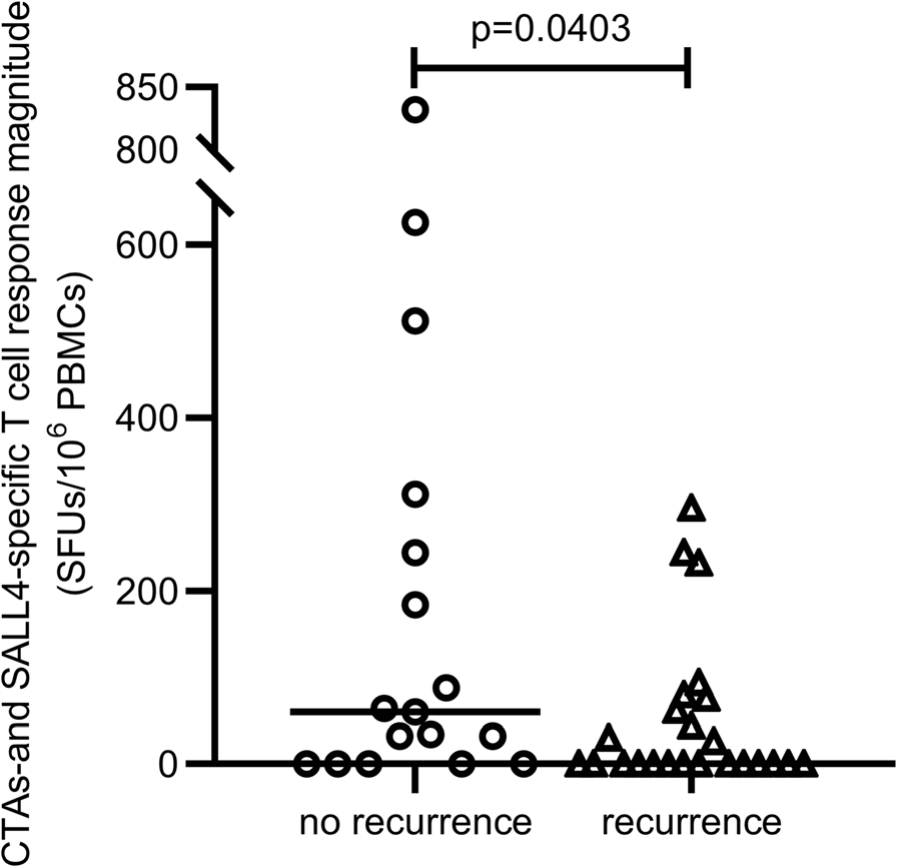
CTA & SALL4-specific T cell response magnitude was analyzed according to 1-year prognosis after TACE + RFA. There were 41 HCC patients (early-stage vs. advanced-stage: 25 vs. 16) that achieved complete ablation effect, and 24 patients developed recurrence while 17 patients among them didn’t get recurrence at 1 year after treatment. Each dot represents one tumour-specific T cell response. Values were compared by Mann-Whitney U-test

Forty-one out of 58 HCC patients received TACE combined with RFA therapy. All 41 patients were evaluated as having complete ablation of their tumours on dynamic contrast enhancement CT scans after the operation. Among them, 17 patients did not experience recurrence of HCC during the one-year follow-up. The correlation between CTA- and SALL4-specific T cell responses and recurrence was analyzed, and a significantly stronger T cell response to tumour antigens was found in patients without recurrence than in those who experienced relapse (177.65 ± 61.21 SFUs/10^6^ cells vs. 49.33 ± 17.60 SFUs/10^6^ cells, *P* = 0.0403, Fig. 4). Patients with early-stage HCC had T cells that could recognize several tumour antigens, and these specific T cells represent protective immunity.

**Fig. 5:**
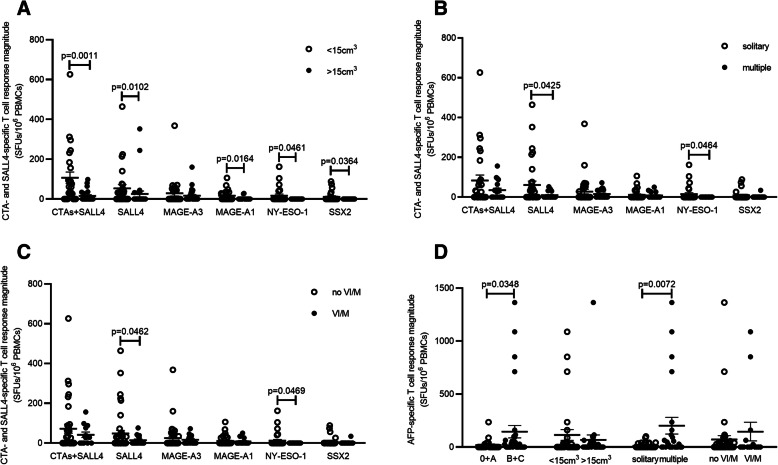
Different antigen specific T cell responses in diverse tumour characteristics. The magnitude of CTA / SALL4-specific T cell response was compared in patients according to their tumour volume (in terms of SALL4 and MAGE-A3, there were 29 subjects in each group; in terms of others, there were 25 subjects with tumour volume < 15 cm^3^) (**A**), tumour number (in terms of SALL4 and MAGE-A3, there were 34 subjects with solitary and 24 with multiple lesions; in terms of others, there were 28 subjects with solitary and 19 with multiple lesions) (**B**), and without or with VI/M (in terms of SALL4 and MAGE-A3, there were 43 subjects without and 15 with VI/M; in terms of others, there were 34 subjects without and 13 with VI/M) (**C**). The comparison of AFP-specific T cell response between patients with different tumour characteristics (there were 26 subjects in 0/A stage and 33 subjects in B/C stage; 29 subjects in each tumour volume group; 34 subjects with solitary and 24 with multiple tumour lesions; 43 subjects without and 15 with VI/M) was presented in (**D**). Data were expressed as mean ± SEM, each dot represents one patient, and values were compared by Mann-Whitney U-test

The magnitude of CTA- and SALL4-specific T cell responses was stronger in patients with a low tumour burden (106.72 ± 29.03 SFUs/10^6^ cells) than in those with a high tumour burden (15.45 ± 6.26 SFUs/10^6^ cells, *P* = 0.0011; Fig. 5A). The frequency of recognition between the two groups was also significantly different [68.97% (20/29) vs. 37.93% (11/29), *P* = 0.018].

Moreover, SALL4- and NY-ESO-1-specific T cell responses in patients with a low tumour burden (53.86 ± 18.3 SFUs/10^6^ cells and 16.88 ± 8.03 SFUs/10^6^ cells, respectively) were significantly higher than those in patients with a high tumour burden (25.59 ± 14.46 SFUs/10^6^ cells and 0, respectively; *P* = 0.0102 and *P* = 0.0461, respectively; Fig. 5A). SALL4- and NY-ESO-1-specific T cell responses were significantly higher in patients with solitary lesions (61 ± 19.1 SFUs/10^6^ cells and 15.07 ± 7.22 SFUs/10^6^ cells, respectively) than in those with multiple lesions (9.58 ± 3.54 SFUs/10^6^ cells, *P* = 0.0425 and 0, *P* = 0.0464, respectively; Fig. 5B). These differences were also present between patients with and without vascular invasion/metastasis (VI/M) (Fig. 5C). In contrast, the AFP-specific T cell response showed correlations with different tumour characteristics opposite to those of the CTA/SALL4-specific T cell response. In particular, in relation to the number of tumour lesions, the AFP-specific T cell response was significantly stronger in patients with multiple lesions (201.75 ± 78.65 SFUs/10^6^ cells) than that in patients with solitary tumour lesions (13.35 ± 4.42 SFUs/10^6^ cells, *P* = 0.0072; Fig. 5D). In addition, a clearly higher AFP-specific T cell response was found in the advanced stage (146.18 ± 58.75 SFUs/10^6^ cells) compared with the early stage (18.88 ± 10.01 SFUs/10^6^ cells; *P* = 0.035; Fig. 5D), which indicated that the AFP-specific T cell response might be a signature of tumour status in the advanced stage. However, we did not find any association between the quantity of serum AFP and the magnitude of the AFP-specific T cell response (data not shown). The AFP-specific T cell immune response could possibly be used as a supplement to serum AFP detection. Patients whose AFP-specific T cell responses are positive or high should be followed up with closely to allow for the early detection of tumours.

### Functional analysis of T cells with the progression of HCC

The restriction of CTA- and TAA-specific T cell responses to CD4+ or CD8+ T cells was analyzed using in vitro culture of T-cell lines stimulated by peptide pools, and the cytokine secretion and degranulation marker CD107a of the MAGE-A3-specific T cell line were evaluated by intracellular cytokine staining (ICS) with TNF-α, IFN-γ, and IL-2. The data shown are from 19 cell lines derived from 5 HCC patients.

**Fig. 6:**
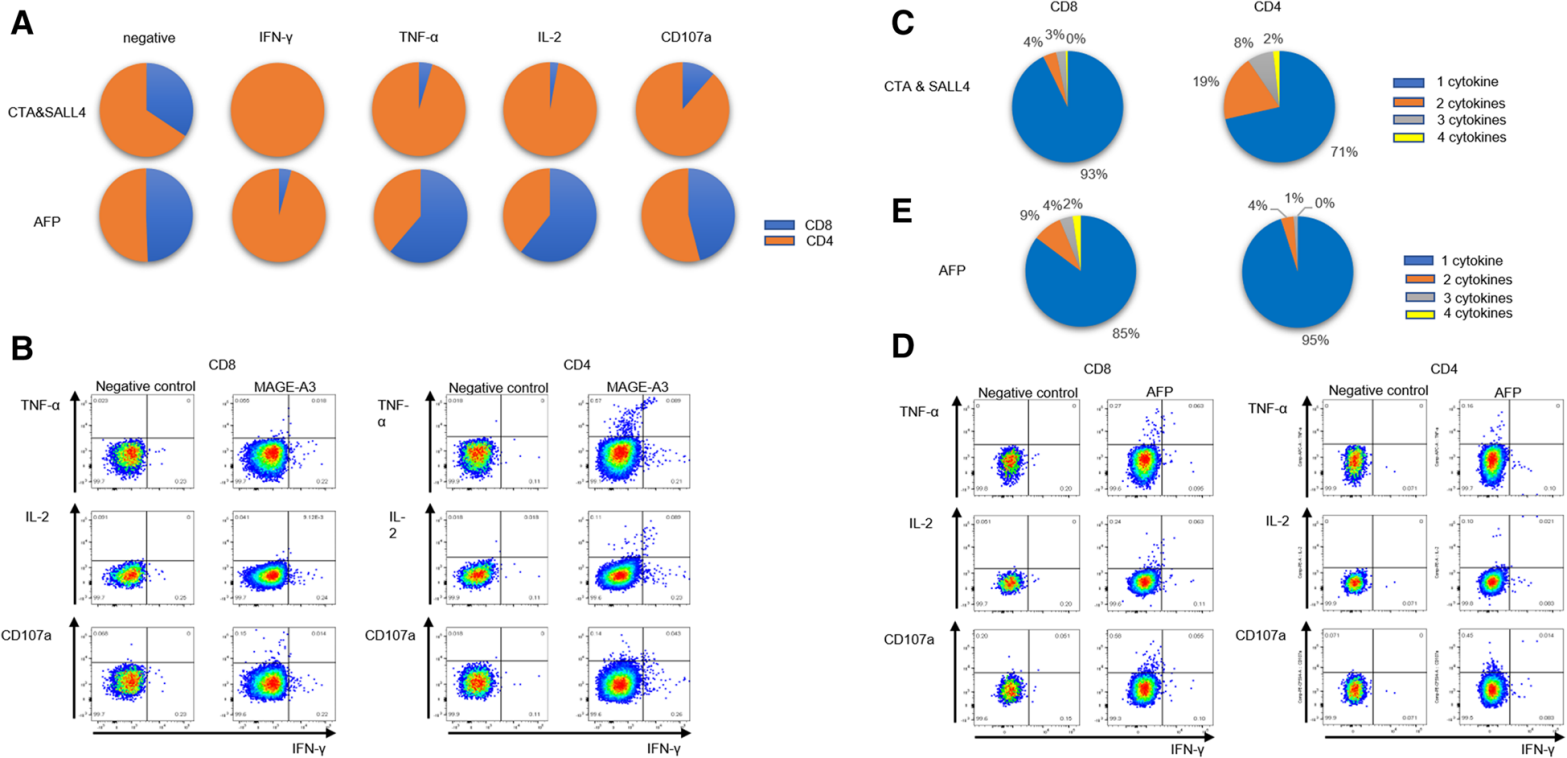
Cytokine profiles of CTA/SALL4 and AFP-specific T cells. The cytokine production of antigen-specific T cells was assessed by ICS after incubation with CTA/SALL4, and AFP (9 PBMC samples were stimulated with CTA / SALL4 and 11 PBMC samples were stimulated with AFP). (**A**) The percentage of CD4+ (orange) and CD8+ (blue) T cells in functional tumour antigen-specific T cells after stimulation of CTA/SALL4, and AFP. The cytokine-secreting and degranulation ability of CD4+ and CD8+ T cells which were generated and stimulated by CTA/SALL4 (**B**) or AFP (**D**). The comparison of proportions of CTA & SALL4-specific (**C**) and AFP-specific (**E**) CD4+ and CD8+ T cells producing one (blue), two (orange), three (grey) and four cytokines (yellow). The data shown are from 19 cell lines derived from 5 HCC patients

A higher proportion of CD4+ T reactive cells was observed, especially CTA/SALL4-specific responses (Fig. 6A). An example ICS is shown in Fig. 6B (The gating strategy was show in Fig. S2). Interestingly, the MAGE-A3-specific T cell population, especially the CD8+ T cell population, was dominated by cells that presented only one functional molecule evaluated (Fig. 6B/C). We also analyzed the T cell phenotype of patients with the AFP-specific T cell response (Fig. 6D/E) (The gating strategy was show in Fig. S3); again, AFP-specific T cell responses were dominated by cells producing one functional molecule evaluated in both the CD4+ and CD8+ T cell populations. Overall, we observed that CTA/SALL4-specific T cell responses are dominated by CD4+ rather than CD8+ T cell responses, with approximately 30% of these cells presenting more than one functional molecule evaluated; in contrast, very few AFP-specific CD4+ T cells present more than one of the functional molecules (5%).

## Discussion

In this study, we found CTA- and TAA-specific T cell responses only in patients with HCC and not in those with LC or CHB. Furthermore, CTA- and TAA-specific T cell responses were detected in 67.24% of HCC patients, which is higher than the rate of serum AFP positivity among patients with HCC [31]. Importantly, we found different hierarchies of CTA- and TAA-specific T cell responses at different stages of HCC. The SALL4-specific T cell response was the strongest, followed by MAGE-A3, NY-ESO-1, MAGE-A1 and SSX2, in early-stage HCC patients, whereas the AFP-specific T cell response was the highest in advanced HCC patients. Two opposite correlations between T cell response and the progression of HCC were identified. This phenomenon indicates the divergence of tumour antigens and that changes in the predominant tumour antigen-specific T cell response in patients with different HCC stages are common. Strong and broad CTA- & SALL4-specific, but not AFP-specific, T cell responses were observed in patients with HCC that was early-stage or less aggressive. A strong relationship between the CTA- and SALL4-specific T cell response and early-stage HCC was identified, suggesting a potential protective role of this T cell response in the partial control of cancer development. Moreover, the association between a high CTA- and SALL4-specific T cell response and a low relapse rate of HCC at the 1-year follow-up further supports the potential protective role of the CTA- and SALL4-specific T cell response in early-stage HCC. Studies on SALL4 showed that the expression of SALL4 was correlated with the malignancy of HCC and suggested a poor prognosis among HCC patients [32, 33]. In this study, patients with early-stage HCC had a stronger SALL4-specific T cell immune response than those with advanced-stage HCC. When HCC progresses to a certain extent, tumour cells expressing SALL4 escape recognition and killing by T cells, and the T cell repertoire changes dramatically at different stages of HCC. Indeed, further study of the relationship between SALL4 expression and the specific T cell response should be performed.

These data, in line with the specific expression of CTA and SALL4 but not AFP in HCC but not normal liver, indicate the potentially important role of the CTA- and SALL4-specific T cell response in the early stage of HCC and in the control of HCC recurrence. Our study further confirmed that the expression of CTA and SALL4 in HCC tumours might promote antitumour immune surveillance and facilitate postoperative recovery [18]. Although CTA DNA is found in the late stage of HCC [34], there is a complex series of processes from gene expression to functional antigen production that can elicit an appropriate protective immune response. For example, the activity of the antigen, the immunogenicity, the expression of MHC molecule, the affinity between the MHC molecule and the antigen, and the ability of the T cell to recognize the antigen and to play a protection role against the antigen-expressing tumour cells. Recently, immunotherapy for HCC [9, 10] has not only provided more powerful evidence to support the role of immune system in controlling HCC progression but has also indicated that our understanding of T cell immune responses to HCC is insufficient, especially our understanding of diverse T cell immune statuses in different stages of HCC. Furthermore, a successfully generated MAGE-A3-specific short-term T cell line showed that specific cytokine-secreting T cells were restricted by CD4+ T cells, not CD8+ T cells. These CD8+ T cells could proliferate but remained impaired with respect to functions and were therefore undetectable by intracellular cytokine staining. Functional T cells can coexist with tumours with persisting antigens if the expression level of tumour antigens or the frequency of the T cells encountering tumour antigens is low [35, 36]. The study of Junliang Fu et al. demonstrated that CD4+ cytotoxic T cells correlated with the survival outcomes of HCC patients [37]. Additionally, further research is needed to reveal the true nature of tumour antigen-specific T cells.

It is known that approximately 50% of HCCs secrete AFP [38, 39], which is not only an oncofoetal antigen and diagnostic marker for liver cancer [40] but also an independent risk predictor associated with pathological grade, progression, and survival outcome [41]. In this study, the AFP-specific T cell response was not only found to be specific to HCC patients but was also highly distributed in patients with advanced-stage HCC, which implied that the AFP-specific T cell response might be a signature of tumour status in the advanced stage. The AFP-specific T cell response is common in patients with advanced-stage disease, which indicates the interaction between the protective role of the host T cell immune response to control the progression of HCC and the various mutations or antigenic drift from tumour cells to escape immune killing, immunoediting and immune surveillance [5, 42, 43]. ICS with a short-term T cell line showed that the AFP-specific T cell response was predominantly restricted by CD8+ T cells, which, as responsive T cells, is a signature of malignant tumour status. We observed that most somatic mutations were tolerated and accumulated neutrally, confirming that mutations generating neoantigens with high immunogenicity are rare in HCC or were already immune-eliminated [42]. The cell-mediated cytotoxicity of specific T cells is impaired early in the cells’ fate [36] in the presence of persistent antigens. Similar to intertumoral heterogeneity, the tumour-antigen-specific T cell response also displayed distinctions among patients, possibly due to different immunogenic stimuli or levels of immune escape. Of course, whether the function of AFP-specific T lymphocytes can be efficiently activated in vivo to target AFP-expressing tumour cells needs to be further explored.

Accordingly, we propose that tumour cells mutate and escape killing by immune cells during the progression of HCC. As tumour cells that express antigens and are recognized and killed by immune cells can be eliminated due to a lack of survival advantages, different antigen recognition spectra appear between patients with HCC in the early and advanced stages. The T-cell immune responses in early-stage HCC patients show diversity, while those with advanced-stage HCC predominantly display AFP-specific T-cell immune responses and few other T-cells that cannot recognize tumour cells as a result of tumour evolution. Survival is achieved only by avoiding recognition and killing by immune cells. In the advanced stage of HCC, AFP-specific T cells become dominant and the main driving force of antitumour immunity. However, the survival ability of tumour cells at this stage has exceeded the killing ability of this antitumour immunity, and solitary AFP-specific T cells cannot control tumour progression. “While the priest climbs a post, the devil climbs ten.” As the main driver of the immune response, AFP-specific T cells are insufficient to control and eliminate the continued growth of the malignant cells, and the ultimate result is tumour progression.

## Conclusions

The results of this study indicated that the CTA- and TAA-specific T cell response was only present in patients with HCC. Different hierarchies of CTA- and TAA-specific T cell responses were found at different stages of HCC. The SALL4-specific T cell response was the strongest response, followed by MAGE-A3, NY-ESO-1, MAGE-A1 and SSX2, in patients with early-stage HCC, whereas the AFP-specific T cell response was the highest in patients with advanced-stage HCC. Furthermore, strong and broad CTA- & SALL4-specific, but not AFP-specific, T cell responses were observed in patients with HCC that was early-stage, less aggressive or with a low relapse rate at the 1-year follow-up. The application of immunotherapy in the early stage of HCC may benefit patients more.

## Supplementary Information

**Additional file 1:** Table S1. The sequences of the overlapping peptides for each of the six antigens and their position within the protein sequence.

**Additional file 2:** Fig. S1. The example picture of the tumour antigen-specific T cell response results in HC, CHB, LC controls and HCC.

**Additional file 3:** Fig. S2. Gating strategy of cytokines on CD4 and CD8 T cells in MAGE-A3-stimulated short-term T cell lines. Progressive gating strategy was used to exclude doublets and dead cells and to identify CD4 and CD8 T cells afterwards. Unstimulated controls were applied accordingly in order to properly position gates of cytokines. The abscissa axis of the final gating strategy graphs was IFN-γ, and the vertical axis of the three gating strategy graphs of CD8+ T cells was TNF-α, IL-2, and CD107a from top to bottom, of CD4+ T cells was TNF-α, IL-2, and CD107a from left to right.

**Additional file 4:** Fig. S3. Gating strategy of cytokines on CD4 and CD8 T cells in AFP-stimulated short-term T cell lines. Progressive gating strategy was used to exclude doublets and dead cells and to identify CD4 and CD8 T cells afterwards. Unstimulated controls were applied accordingly in order to properly position gates of cytokines. The abscissa axis of the final gating strategy graphs was IFN-γ, and the vertical axis of the three gating strategy graphs of CD8+ T cells was TNF-α, IL-2, and CD107a from top to bottom, of CD4+ T cells was TNF-α, IL-2, and CD107a from left to right.

## Data Availability

The raw data of this study are derived from our hospital. All detailed data included in the study are available upon request by contact with the corresponding author.

## Abbreviations

AFP: Alpha-fetoprotein
BCLC: Barcelona Clinic Liver Cancer staging system
CHB: Chronic Hepatitis B
CTA: Cancer testis antigen
ELISPOT: Enzyme-linked immunospot assay
HC: Healthy control
HCC: Hepatocellular carcinoma
ICS: Intracellular cytokine staining
IFN-γ: Interferon-γ
IL-2: Interleukin-2
LC: Liver cirrhosis
MAGE-A1: Melanoma-associated antigen A1
NY-ESO-1: New York esophageal squamous cell carcinoma antigen 1
RFA: Radiofrequency ablation
SALL4: Sal-like gene 4
SFU: Spot-forming unit
SSX2: Synovial sarcoma X break point gene 2
TAA: Tumor-associated-antigen
TACE: Transcatheter arterial chemoembolization
TNF-α: Tumor necrosis factor α
VI/M: Vascular invasion/metastasis
