# Supplementary material for: Distinct tumour antigen-specific T-cell immune response profiles at different hepatocellular carcinoma stages
Source: BMC Cancer. 2021 Sep 8;21:1007. doi: 10.1186/s12885-021-08720-9 (PMC8428121; doi:10.1186/s12885-021-08720-9)
Supplement: Supplementary file 1 — Additional file 1: Table S1. The sequences of the overlapping peptides for each of the six antigens and their position within the protein sequence. [file 12885_2021_8720_MOESM1_ESM.docx]

**Supplementary Table 1**

The sequences of the overlapping peptides for each of the six antigens and their position within the protein sequence are shown.

**List of overlapping peptides**

| **peptide** | **sequence** |
| --- | --- |
| AFP-1 | MKWVESIFLIFLLNFTES |
| AFP-2 | LIFLLNFTESRTLHRNEY |
| AFP-3 | ESRTLHRNEYGIASILDS |
| AFP-4 | EYGIASILDSYQCTAEIS |
| AFP-5 | DSYQCTAEISLADLATIF |
| AFP-6 | ISLADLATIFFAQFVQEA |
| AFP-7 | IFFAQFVQEATYKEVSKM |
| AFP-8 | EATYKEVSKMVKDALTAI |
| AFP-9 | KMVKDALTAIEKPTGDEQ |
| AFP-10 | AIEKPTGDEQSSGCLENQ |
| AFP-11 | EQSSGCLENQLPAFLEEL |
| AFP-12 | NQLPAFLEELCHEKEILE |
| AFP-13 | ELCHEKEILEKYGHSDCC |
| AFP-14 | LEKYGHSDCCSQSEEGRH |
| AFP-15 | CCSQSEEGRHNCFLAHKK |
| AFP-16 | RHNCFLAHKKPTPASIPL |
| AFP-17 | KKPTPASIPLFQVPEPVT |
| AFP-18 | PLFQVPEPVTSCEAYEED |
| AFP-19 | VTSCEAYEEDRETFMNKF |
| AFP-20 | EDRETFMNKFIYEIARRH |
| AFP-21 | KFIYEIARRHPFLYAPTI |
| AFP-22 | RHPFLYAPTILLWAARYD |
| AFP-23 | TILLWAARYDKIIPSCCK |
| AFP-24 | YDKIIPSCCKAENAVECF |
| AFP-25 | CKAENAVECFQTKAATVT |
| AFP-26 | CFQTKAATVTKELRESSL |
| AFP-27 | VTKELRESSLLNQHACAV |
| AFP-28 | SLLNQHACAVMKNFGTRT |
| AFP-29 | AVMKNFGTRTFQAITVTK |
| AFP-30 | RTFQAITVTKLSQKFTKV |
| AFP-31 | TKLSQKFTKVNFTEIQKL |
| AFP-32 | KVNFTEIQKLVLDVAHVH |
| AFP-33 | KLVLDVAHVHEHCCRGDV |
| AFP-34 | VHEHCCRGDVLDCLQDGE |
| AFP-35 | DVLDCLQDGEKIMSYICS |
| AFP-36 | GEKIMSYICSQQDTLSNK |
| AFP-37 | CSQQDTLSNKITECCKLT |
| AFP-38 | NKITECCKLTTLERGQCI |
| AFP-39 | LTTLERGQCIIHAENDEK |
| AFP-40 | CIIHAENDEKPEGLSPNL |
| AFP-41 | EKPEGLSPNLNRFLGDRD |
| AFP-42 | NLNRFLGDRDFNQFSSGE |
| AFP-43 | RDFNQFSSGEKNIFLASF |
| AFP-44 | GEKNIFLASFVHEYSRRH |
| AFP-45 | SFVHEYSRRHPQLAVSVI |
| AFP-46 | RHPQLAVSVILRVAKGYQ |
| AFP-47 | VILRVAKGYQELLEKCFQ |
| AFP-48 | YQELLEKCFQTENPLECQ |
| AFP-49 | FQTENPLECQDKGEEELQ |
| AFP-50 | CQDKGEEELQKYIQESQA |
| AFP-51 | LQKYIQESQALAKRSCGL |
| AFP-52 | QALAKRSCGLFQKLGEYY |
| AFP-53 | GLFQKLGEYYLQNAFLVA |
| AFP-54 | YYLQNAFLVAYTKKAPQL |
| AFP-55 | VAYTKKAPQLTSSELMAI |
| AFP-56 | QLTSSELMAITRKMAATA |
| AFP-57 | AITRKMAATAATCCQLSE |
| AFP-58 | TAATCCQLSEDKLLACGE |
| AFP-59 | SEDKLLACGEGAADIIIG |
| AFP-60 | GEGAADIIIGHLCIRHEM |
| AFP-61 | IGHLCIRHEMTPVNPGVG |
| AFP-62 | EMTPVNPGVGQCCTSSYA |
| AFP-63 | VGQCCTSSYANRRPCFSS |
| AFP-64 | YANRRPCFSSLVVDETYV |
| AFP-65 | SSLVVDETYVPPAFSDDK |
| AFP-66 | YVPPAFSDDKFIFHKDLC |
| AFP-67 | DKFIFHKDLCQAQGVALQ |
| AFP-68 | LCQAQGVALQTMKQEFLI |
| AFP-69 | LQTMKQEFLINLVKQKPQ |
| AFP-70 | LINLVKQKPQITEEQLEA |
| AFP-71 | PQITEEQLEAVIADFSGL |
| AFP-72 | EAVIADFSGLLEKCCQGQ |
| AFP-73 | GLLEKCCQGQEQEVCFAE |
| AFP-74 | GQEQEVCFAEEGQKLISK |
| AFP-75 | AEEGQKLISKTRAALGV |
| SALL-4-1 | MSRRKQAKPQHINSEEDQ |
| SALL-4-2 | PQHINSEEDQGEQQPQQQ |
| SALL-4-3 | DQGEQQPQQQTPEFADAA |
| SALL-4-4 | QQTPEFADAAPAAPAAGE |
| SALL-4-5 | AAPAAPAAGELGAPVNHP |
| SALL-4-6 | GELGAPVNHPGNDEVASE |
| SALL-4-7 | HPGNDEVASEDEATVKRL |
| SALL-4-8 | SEDEATVKRLRREETHVC |
| SALL-4-9 | RLRREETHVCEKCCAEFF |
| SALL-4-10 | VCEKCCAEFFSISEFLEH |
| SALL-4-11 | FFSISEFLEHKKNCTKNP |
| SALL-4-12 | EHKKNCTKNPPVLIMNDS |
| SALL-4-13 | NPPVLIMNDSEGPVPSED |
| SALL-4-14 | DSEGPVPSEDFSGAVLSH |
| SALL-4-15 | EDFSGAVLSHQPTSPGSK |
| SALL-4-16 | SHQPTSPGSKDCHRENGG |
| SALL-4-17 | SKDCHRENGGSSEDMKEK |
| SALL-4-18 | GGSSEDMKEKPDAESVVY |
| SALL-4-19 | EKPDAESVVYLKTETALP |
| SALL-4-20 | VYLKTETALPPTPQDISY |
| SALL-4-21 | LPPTPQDISYLAKGKVAN |
| SALL-4-22 | SYLAKGKVANTNVTLQAL |
| SALL-4-23 | ANTNVTLQALRGTKVAVN |
| SALL-4-24 | ALRGTKVAVNQRSADALP |
| SALL-4-25 | VNQRSADALPAPVPGANS |
| SALL-4-26 | LPAPVPGANSIPWVLEQI |
| SALL-4-27 | NSIPWVLEQILCLQQQQL |
| SALL-4-28 | QILCLQQQQLQQIQLTEQ |
| SALL-4-29 | QLQQIQLTEQIRIQVNMW |
| SALL-4-30 | EQIRIQVNMWASHALHSS |
| SALL-4-31 | MWASHALHSSGAGADTLK |
| SALL-4-32 | SSGAGADTLKTLGSHMSQ |
| SALL-4-33 | LKTLGSHMSQQVSAAVAL |
| SALL-4-34 | SQQVSAAVALLSQKAGSQ |
| SALL-4-35 | ALLSQKAGSQGLSLDALK |
| SALL-4-36 | SQGLSLDALKQAKLPHAN |
| SALL-4-37 | LKQAKLPHANIPSATSSL |
| SALL-4-38 | ANIPSATSSLSPGLAPFT |
| SALL-4-39 | SLSPGLAPFTLKPDGTRV |
| SALL-4-40 | FTLKPDGTRVLPNVMSRL |
| SALL-4-41 | RVLPNVMSRLPSALLPQA |
| SALL-4-42 | RLPSALLPQAPGSVLFQS |
| SALL-4-43 | QAPGSVLFQSPFSTVALD |
| SALL-4-44 | QSPFSTVALDTSKKGKGK |
| SALL-4-45 | LDTSKKGKGKPPNISAVD |
| SALL-4-46 | GKPPNISAVDVKPKDEAA |
| SALL-4-47 | VDVKPKDEAALYKHKCKY |
| SALL-4-48 | AALYKHKCKYCSKVFGTD |
| SALL-4-49 | KYCSKVFGTDSSLQIHLR |
| SALL-4-50 | TDSSLQIHLRSHTGERPF |
| SALL-4-51 | LRSHTGERPFVCSVCGHR |
| SALL-4-52 | PFVCSVCGHRFTTKGNLK |
| SALL-4-57 | VAAGNGIPYALSVPDPID |
| SALL-4-58 | YALSVPDPIDEPSLSLDS |
| SALL-4-59 | IDEPSLSLDSKPVLVTTS |
| SALL-4-60 | DSKPVLVTTSVGLPQNLS |
| SALL-4-61 | TSVGLPQNLSSGTNPKDL |
| SALL-4-62 | LSSGTNPKDLTGGSLPGD |
| SALL-4-63 | DLTGGSLPGDLQPGPSPE |
| SALL-4-64 | GDLQPGPSPESEGGPTLP |
| SALL-4-65 | PESEGGPTLPGVGPNYNS |
| SALL-4-66 | LPGVGPNYNSPRAGGFQG |
| SALL-4-67 | NSPRAGGFQGSGTPEPGS |
| SALL-4-68 | QGSGTPEPGSETLKLQQL |
| SALL-4-69 | GSETLKLQQLVENIDKAT |
| SALL-4-70 | QLVENIDKATTDPNECLI |
| SALL-4-71 | ATTDPNECLICHRVLSCQ |
| SALL-4-72 | LICHRVLSCQSSLKMHYR |
| SALL-4-73 | CQSSLKMHYRTHTGERPF |
| SALL-4-74 | YRTHTGERPFQCKICGRA |
| SALL-4-75 | PFQCKICGRAFSTKGNLK |
| SALL-4-76 | RAFSTKGNLKTHLGVHRT |
| SALL-4-77 | LKTHLGVHRTNTSIKTQH |
| SALL-4-78 | RTNTSIKTQHSCPICQKK |
| SALL-4-79 | QHSCPICQKKFTNAVMLQ |
| SALL-4-80 | KKFTNAVMLQQHIRMHMG |
| SALL-4-81 | LQQHIRMHMGGQIPNTPL |
| SALL-4-82 | MGGQIPNTPLPENPCDFT |
| SALL-4-83 | PLPENPCDFTGSEPMTVG |
| SALL-4-84 | FTGSEPMTVGENGSTGAI |
| SALL-4-85 | VGENGSTGAICHDDVIES |
| SALL-4-86 | AICHDDVIESIDVEEVSS |
| SALL-4-87 | ESIDVEEVSSQEAPSSSS |
| SALL-4-88 | SSQEAPSSSSKVPTPLPS |
| SALL-4-89 | SSKVPTPLPSIHSASPTL |
| SALL-4-90 | PSIHSASPTLGFAMMASL |
| SALL-4-91 | TLGFAMMASLDAPGKVGP |
| SALL-4-92 | SLDAPGKVGPAPFNLQRQ |
| SALL-4-93 | GPAPFNLQRQGSRENGSV |
| SALL-4-94 | RQGSRENGSVESDGLTND |
| SALL-4-95 | SVESDGLTNDSSSLMGDQ |
| SALL-4-96 | NDSSSLMGDQEYQSRSPD |
| SALL-4-97 | DQEYQSRSPDILETTSFQ |
| SALL-4-98 | PDILETTSFQALSPANSQ |
| SALL-4-99 | FQALSPANSQAESIKSKS |
| SALL-4-100 | SQAESIKSKSPDAGSKAE |
| SALL-4-101 | KSPDAGSKAESSENSRTE |
| SALL-4-102 | AESSENSRTEMEGRSSLP |
| SALL-4-103 | TEMEGRSSLPSTFIRAPP |
| SALL-4-104 | LPSTFIRAPPTYVKVEVP |
| SALL-4-105 | PPTYVKVEVPGTFVGPST |
| SALL-4-106 | VPGTFVGPSTLSPGMTPL |
| SALL-4-107 | STLSPGMTPLLAAQPRRQ |
| SALL-4-108 | PLLAAQPRRQAKQHGCTR |
| SALL-4-109 | RQAKQHGCTRCGKNFSSA |
| SALL-4-110 | TRCGKNFSSASALQIHER |
| SALL-4-111 | SASALQIHERTHTGEKPF |
| SALL-4-112 | ERTHTGEKPFVCNICGRA |
| SALL-4-113 | PFVCNICGRAFTTKGNLK |
| SALL-4-114 | RAFTTKGNLKVHYMTHGA |
| SALL-4-115 | LKVHYMTHGANNNSARRG |
| SALL-4-116 | GANNNSARRGRKLAIENT |
| SALL-4-117 | RGRKLAIENTMALLGTDG |
| SALL-4-118 | NTMALLGTDGKRVSEIFP |
| SALL-4-119 | DGKRVSEIFPKEILAPSV |
| SALL-4-120 | FPKEILAPSVNVDPVVWN |
| SALL-4-121 | SVNVDPVVWNQYTSMLNG |
| SALL-4-122 | WNQYTSMLNGGLAVKTNE |
| SALL-4-123 | NGGLAVKTNEISVIQSGG |
| SALL-4-124 | NEISVIQSGGVPTLPVSL |
| SALL-4-125 | GGVPTLPVSLGATSVVNN |
| SALL-4-126 | SLGATSVVNNATVSKMDG |
| SALL-4-127 | NNATVSKMDGSQSGISAD |
| SALL-4-128 | DGSQSGISADVEKPSATD |
| SALL-4-129 | ADVEKPSATDGVPKHQFP |
| SALL-4-130 | TDGVPKHQFPHFLEENKI |
| SALL-4-131 | FPHFLEENKIAVS |
| MAGE-A3-1 | MPLEQRSQHCKPEEGLEA |
| MAGE-A3-2 | HCKPEEGLEARGEALGLV |
| MAGE-A3-3 | EARGEALGLVGAQAPATE |
| MAGE-A3-4 | LVGAQAPATEEQEAASSS |
| MAGE-A3-5 | TEEQEAASSSSTLVEVTL |
| MAGE-A3-6 | SSSTLVEVTLGEVPAAES |
| MAGE-A3-7 | TLGEVPAAESPDPPQSPQ |
| MAGE-A3-8 | ESPDPPQSPQGASSLPTT |
| MAGE-A3-9 | PQGASSLPTTMNYPLWSQ |
| MAGE-A3-10 | TTMNYPLWSQSYEDSSNQ |
| MAGE-A3-11 | SQSYEDSSNQEEEGPSTF |
| MAGE-A3-12 | NQEEEGPSTFPDLESEFQ |
| MAGE-A3-13 | TFPDLESEFQAALSRKVA |
| MAGE-A3-14 | FQAALSRKVAELVHFLLL |
| MAGE-A3-15 | VAELVHFLLLKYRAREPV |
| MAGE-A3-16 | LLKYRAREPVTKAEMLGS |
| MAGE-A3-17 | PVTKAEMLGSVVGNWQYF |
| MAGE-A3-18 | GSVVGNWQYFFPVIFSKA |
| MAGE-A3-19 | YFFPVIFSKASSSLQLVF |
| MAGE-A3-20 | KASSSLQLVFGIELMEVD |
| MAGE-A3-21 | VFGIELMEVDPIGHLYIF |
| MAGE-A3-22 | VDPIGHLYIFATCLGLSY |
| MAGE-A3-23 | IFATCLGLSYDGLLGDNQ |
| MAGE-A3-24 | SYDGLLGDNQIMPKAGLL |
| MAGE-A3-25 | NQIMPKAGLLIIVLAIIA |
| MAGE-A3-26 | LLIIVLAIIAREGDCAPE |
| MAGE-A3-27 | IAREGDCAPEEKIWEELS |
| MAGE-A3-28 | PEEKIWEELSVLEVFEGR |
| MAGE-A3-29 | LSVLEVFEGREDSILGDP |
| MAGE-A3-30 | GREDSILGDPKKLLTQHF |
| MAGE-A3-31 | DPKKLLTQHFVQENYLEY |
| MAGE-A3-32 | HFVQENYLEYRQVPGSDP |
| MAGE-A3-33 | EYRQVPGSDPACYEFLWG |
| MAGE-A3-34 | DPACYEFLWGPRALVETS |
| MAGE-A3-35 | WGPRALVETSYVKVLHHM |
| MAGE-A3-36 | TSYVKVLHHMVKISGGPH |
| MAGE-A3-37 | HMVKISGGPHISYPPLHE |
| MAGE-A3-38 | PHISYPPLHEWVLREGEE |
| MAGE-A3-39 | HEWVLREGEE |
| MAGE-A1-1 | MSLEQRSLHCKPEEALEA |
| MAGE-A1-2 | HCKPEEALEAQQEALGLV |
| MAGE-A1-3 | EAQQEALGLVCVQAATSS |
| MAGE-A1-4 | LVCVQAATSSSSPLVLGT |
| MAGE-A1-5 | SSSSPLVLGTLEEVPTAG |
| MAGE-A1-6 | GTLEEVPTAGSTDPPQSP |
| MAGE-A1-7 | AGSTDPPQSPQGASAFPT |
| MAGE-A1-8 | SPQGASAFPTTINFTRQR |
| MAGE-A1-9 | PTTINFTRQRQPSEGSSS |
| MAGE-A1-10 | QRQPSEGSSSREEEGPST |
| MAGE-A1-11 | SSREEEGPSTSCILESLF |
| MAGE-A1-12 | STSCILESLFRAVITKKV |
| MAGE-A1-13 | LFRAVITKKVADLVGFLL |
| MAGE-A1-14 | KVADLVGFLLLKYRAREP |
| MAGE-A1-15 | LLLKYRAREPVTKAEMLE |
| MAGE-A1-16 | EPVTKAEMLESVIKNYKH |
| MAGE-A1-17 | LESVIKNYKHCFPEIFGK |
| MAGE-A1-18 | KHCFPEIFGKASESLQLV |
| MAGE-A1-19 | GKASESLQLVFGIDVKEA |
| MAGE-A1-20 | LVFGIDVKEADPTGHSYV |
| MAGE-A1-21 | EADPTGHSYVLVTCLGLS |
| MAGE-A1-22 | YVLVTCLGLSYDGLLGDN |
| MAGE-A1-23 | LSYDGLLGDNQIMPKTGF |
| MAGE-A1-24 | DNQIMPKTGFLIIVLVMA |
| MAGE-A1-25 | GFLIIVLVMAMEGGHAPE |
| MAGE-A1-26 | MAMEGGHAPEEEIWEELS |
| MAGE-A1-27 | PEEEIWEELSVMEVYDGR |
| MAGE-A1-28 | LSVMEVYDGREHSAYGEP |
| MAGE-A1-29 | GREHSAYGEPRKLLTQDL |
| MAGE-A1-30 | EPRKLLTQDLVQEKYLEY |
| MAGE-A1-31 | DLVQEKYLEYRQVPDSDP |
| MAGE-A1-32 | EYRQVPDSDPARYEFLWG |
| MAGE-A1-33 | DPARYEFLWGPRALAETS |
| MAGE-A1-34 | WGPRALAETSYVKVLEYV |
| MAGE-A1-35 | TSYVKVLEYVIKVSARVR |
| MAGE-A1-36 | YVIKVSARVRFFFPSLRE |
| MAGE-A1-37 | VRFFFPSLREAALREEEE |
| MAGE-A1-38 | REAALREEEEGVMSLEQR |
| MAGE-A1-39 | EEGVMSLEQRSLHCKPEE |
| NY-ESO-1-1 | MQAEGRGTGGSTGDADGP |
| NY-ESO-1-2 | GGSTGDADGPGGPGIPDG |
| NY-ESO-1-3 | GPGGPGIPDGPGGNAGGP |
| NY-ESO-1-4 | DGPGGNAGGPGEAGATGG |
| NY-ESO-1-5 | GPGEAGATGGRGPRGAGA |
| NY-ESO-1-6 | GGRGPRGAGAARASGPGG |
| NY-ESO-1-7 | GAARASGPGGGAPRGPHG |
| NY-ESO-1-8 | GGGAPRGPHGGAASGLNG |
| NY-ESO-1-9 | HGGAASGLNGCCRCGARG |
| NY-ESO-1-10 | NGCCRCGARGPESRLLEF |
| NY-ESO-1-11 | RGPESRLLEFYLAMPFAT |
| NY-ESO-1-12 | EFYLAMPFATPMEAELAR |
| NY-ESO-1-13 | ATPMEAELARRSLAQDAP |
| NY-ESO-1-14 | ARRSLAQDAPPLPVPGVL |
| NY-ESO-1-15 | APPLPVPGVLLKEFTVSG |
| NY-ESO-1-16 | VLLKEFTVSGNILTIRLT |
| NY-ESO-1-17 | SGNILTIRLTAADHRQLQ |
| NY-ESO-1-18 | LTAADHRQLQLSISSCLQ |
| NY-ESO-1-19 | LQLSISSCLQQLSLLMWI |
| NY-ESO-1-20 | LQQLSLLMWITQCFLPVF |
| NY-ESO-1-21 | WITQCFLPVFLAQPPSGQ |
| NY-ESO-1-22 | VFLAQPPSGQRRMQAEGR |
| NY-ESO-1-23 | GQRRMQAEGRGTGGSTGD |
| SSX-2-1 | MNGDDAFARRPTVGAQIP |
| SSX-2-2 | RRPTVGAQIPEKIQKAFD |
| SSX-2-3 | IPEKIQKAFDDIAKYFSK |
| SSX-2-4 | FDDIAKYFSKEEWEKMKA |
| SSX-2-5 | SKEEWEKMKASEKIFYVY |
| SSX-2-6 | KASEKIFYVYMKRKYEAM |
| SSX-2-7 | VYMKRKYEAMTKLGFKAT |
| SSX-2-8 | AMTKLGFKATLPPFMCNK |
| SSX-2-9 | ATLPPFMCNKRAEDFQGN |
| SSX-2-10 | NKRAEDFQGNDLDNDPNR |
| SSX-2-11 | GNDLDNDPNRGNQVERPQ |
| SSX-2-12 | NRGNQVERPQMTFGRLQG |
| SSX-2-13 | PQMTFGRLQGISPKIMPK |
| SSX-2-14 | QGISPKIMPKKPAEEGND |
| SSX-2-15 | PKKPAEEGNDSEEVPEAS |
| SSX-2-16 | NDSEEVPEASGPQNDGKE |
| SSX-2-17 | ASGPQNDGKELCPPGKPT |
| SSX-2-18 | KELCPPGKPTTSEKIHER |
| SSX-2-19 | PTTSEKIHERSGNREAQE |
| SSX-2-20 | ERSGNREAQEKEERRGTA |
| SSX-2-21 | QEKEERRGTAHRWSSQNT |
| SSX-2-22 | TAHRWSSQNTHNIGRFSL |
| SSX-2-23 | NTHNIGRFSLSTSMGAVH |
| SSX-2-24 | SLSTSMGAVHGTPKTITH |
| SSX-2-25 | VHGTPKTITHNRDPKGGN |
| SSX-2-26 | THNRDPKGGNMPGPTDCV |
| SSX-2-27 | GNMPGPTDCVRENSW |

**The design of the nine pools:**

Pool 1: SALL4 _1-45_

Pool 2: SALL4 _46-90_

Pool 3: SALL4 _91-131_

Pool 4: MAGE-A1 _1-39_

Pool 5: MAGE-A3 _1-39_

Pool 6: NY-ESO-1 _1-23_

Pool 7: SSX2 _1-27_

Pool 8: AFP _1-40_

Pool 9: AFP _41-75_
