# Supplementary figures and images for: Distinct tumour antigen-specific T-cell immune response profiles at different hepatocellular carcinoma stages
Source: BMC Cancer. 2021 Sep 8;21:1007. doi: 10.1186/s12885-021-08720-9 (PMC8428121; doi:10.1186/s12885-021-08720-9)

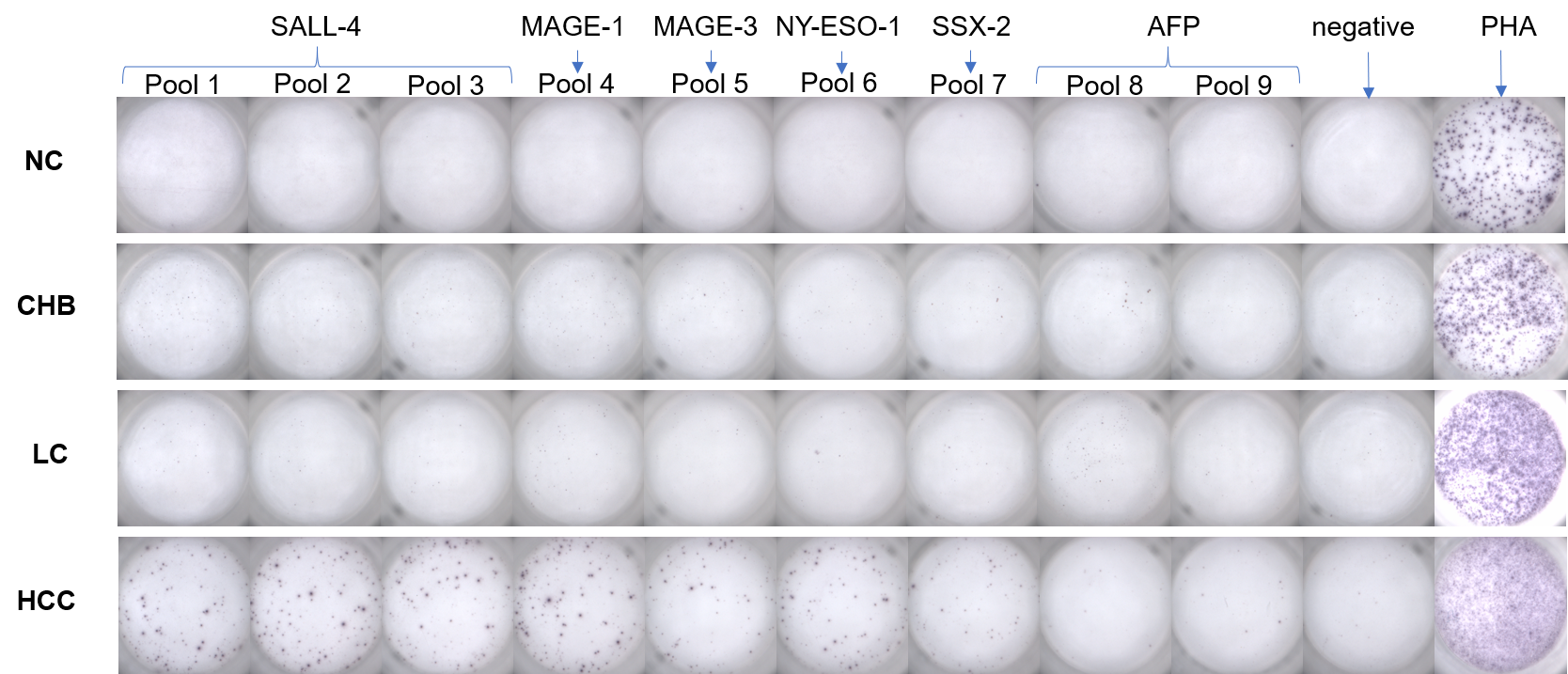

Supplement: Supplementary file 2 — Additional file 2: Fig. S1. The example picture of the tumour antigen-specific T cell response results in HC, CHB, LC controls and HCC. [file 12885_2021_8720_MOESM2_ESM.tif]

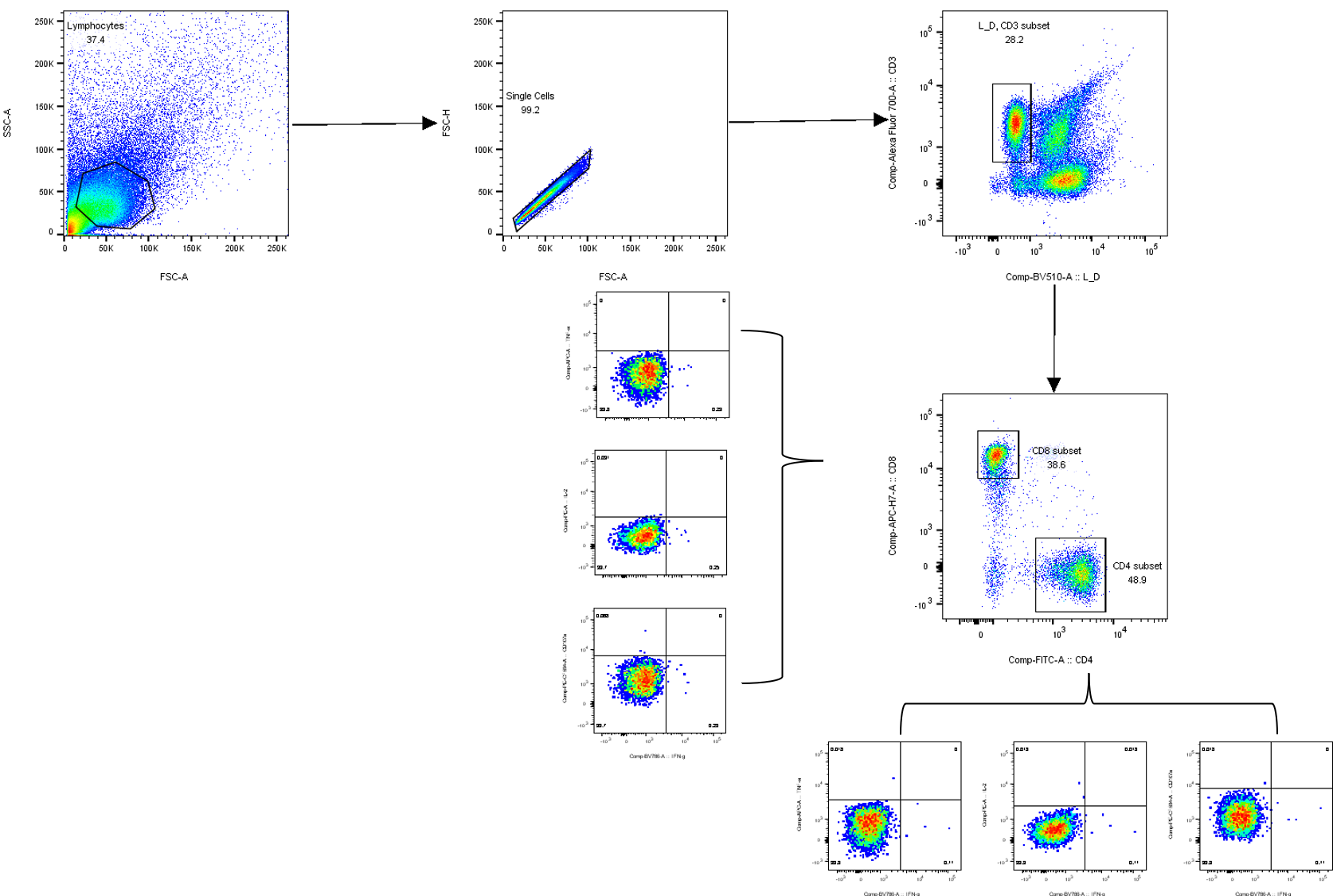

Supplement: Supplementary file 3 — Additional file 3: Fig. S2. Gating strategy of cytokines on CD4 and CD8 T cells in MAGE-A3-stimulated short-term T cell lines. Progressive gating strategy was used to exclude doublets and dead cells and to identify CD4 and CD8 T cells afterwards. Unstimulated controls were applied accordingly in order to properly position gates of cytokines. The abscissa axis of the final gating strategy graphs was IFN-γ, and the vertical axis of the three gating strategy graphs of CD8+ T cells was TNF-α, IL-2, and CD107a from top to bottom, of CD4+ T cells was TNF-α, IL-2, and CD107a from left to right. [file 12885_2021_8720_MOESM3_ESM.tif]

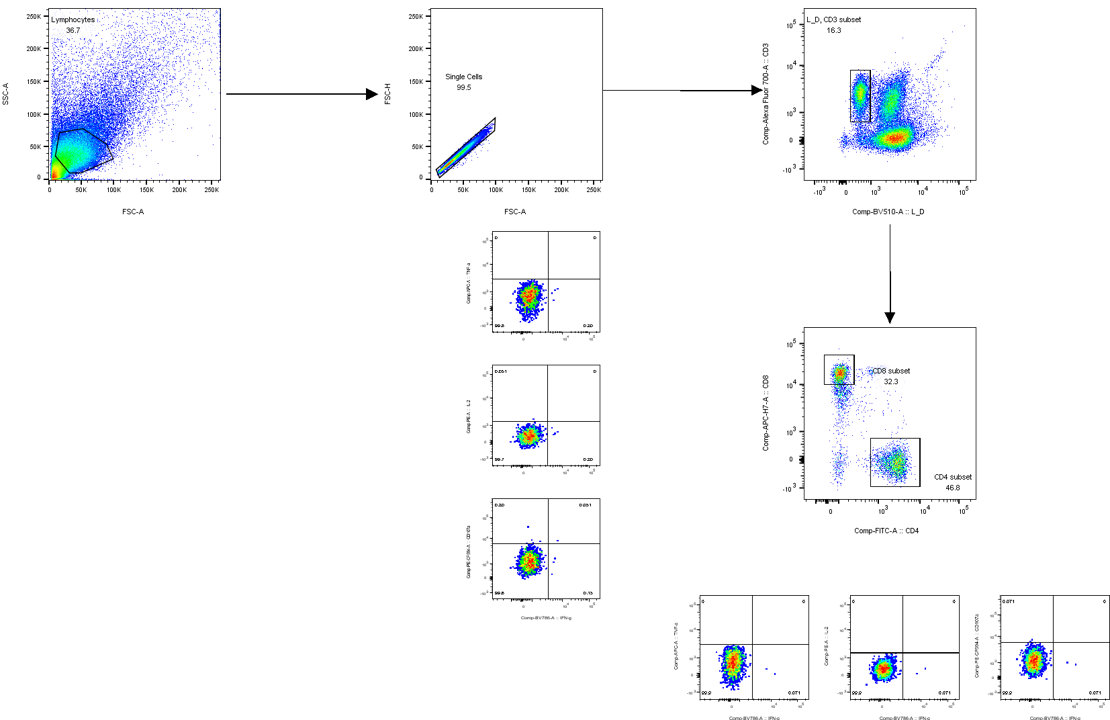

Supplement: Supplementary file 4 — Additional file 4: Fig. S3. Gating strategy of cytokines on CD4 and CD8 T cells in AFP-stimulated short-term T cell lines. Progressive gating strategy was used to exclude doublets and dead cells and to identify CD4 and CD8 T cells afterwards. Unstimulated controls were applied accordingly in order to properly position gates of cytokines. The abscissa axis of the final gating strategy graphs was IFN-γ, and the vertical axis of the three gating strategy graphs of CD8+ T cells was TNF-α, IL-2, and CD107a from top to bottom, of CD4+ T cells was TNF-α, IL-2, and CD107a from left to right. [file 12885_2021_8720_MOESM4_ESM.tif]
